# Validation of a Commercial Indirect ELISA Kit for the Detection of *Bovine alphaherpesvirus* *1* (BoHV-1)-Specific Glycoprotein E Antibodies in Bulk Milk Samples of Dairy Cows

**DOI:** 10.3390/vetsci9070311

**Published:** 2022-06-22

**Authors:** Cecilia Righi, Carmen Iscaro, Laura Ferroni, Sergio Rosati, Claudia Pellegrini, Chiara Nogarol, Elisabetta Rossi, Annalisa Dettori, Francesco Feliziani, Stefano Petrini

**Affiliations:** 1 National Reference Centre for Infectious Bovine Rhinotracheitis (IBR), Istituto Zooprofilattico Sperimentale Umbria-Marche, “Togo Rosati”, 06126 Perugia, Italy; c.iscaro@izsum.it (C.I.); l.ferroni@izsum.it (L.F.); c.pellegrini@izsum.it (C.P.); e.rossi@izsum.it (E.R.); a.dettori@izsum.it (A.D.); f.feliziani@izsum.it (F.F.); s.petrini@izsum.it (S.P.); 2 Department of Veterinary Science, University of Torino, 10124 Torino, Italy; sergio.rosati@unito.it; 3 In3diagnostic srl, Largo P. Braccini 2, 10095 Grugliasco, Italy; chiara.nogarol@in3diagnostic.com

**Keywords:** BoHV-1, gE-ELISA, bulk milk, kit validation

## Abstract

**Simple Summary:**

Recent studies have assessed the feasibility of using commercial indirect enzyme-linked immunosorbent assays (ELISA) on bulk milk (BM) samples. Today is a need to validate a diagnostic method for granting and maintaining a correct surveillance strategy for infectious bovine rhinotracheitis (IBR). However, the poor availability of reference materials for diagnostic tests on milk samples and, the low sensitivity of blocking glycoprotein E (gE) ELISAs using milk as a matrix, represent limitations for developing new assays to control infectious diseases in livestock. This study aimed to validate a commercial indirect ELISA kit to detect antibodies to gE of *Bovine alphaherpesvirus 1* (BoHV-1) from BM samples, according to the OIE Manual of Diagnostic Tests and Vaccines for Terrestrial Animals. The samples were collected from an IBR outbreak. The findings demonstrated high diagnostic performances. Furthermore, the validated kit is an easy-to-use and economical method to discriminate between BoHV-1 infected or immunised animals with gE-deleted markers vaccines using BM samples. Moreover, milk sampling represents a fast and non-invasive procedure for animals because it avoids negative effects, such as stress, and reduces the total cost of surveillance.

**Abstract:**

In this study, we validated a commercial indirect enzyme-linked immunosorbent assay (ELISA) to detect antibodies to glycoprotein E (gE) of *Bovine alphaherpesvirus 1* (BoHV-1) in bulk milk (BM) samples using the OIE Manual of Diagnostic Tests and Vaccines for Terrestrial Animals. The assay performance characteristics were evaluated using a panel of positive (n = 36) and negative (n = 80) samples with known infectious bovine rhinotracheitis (IBR) status. The assay showed adequate repeatability (within-run and between-run), with a coefficient of variability (CV%) of replicates below 30%; only two 1:40 diluted samples had a CV% above 20%. Additionally, an agreement analysis of the qualitative results of replicates led to a Gwet’s agreement coefficient of 0.99 (95% confidence interval (CI): 0.96–1.00, *p* < 0.001). The estimated diagnostic sensitivity (DSe) and diagnostic specificity (DSp) were 100% (95% CI: 90.3–100%) and 97.5% (95% CI: 91.3–99.7%), respectively. Overall, a good level of agreement was observed between the assay results and the true IBR status of samples (weighted Cohen’s κ: 0.96, 95% CI: 0.78–1.00). The findings demonstrate that the indirect ELISA kit validated here is an easy-to-use and economical method to differentiate infected and gE-deleted marker vaccine-immunised animals using BM samples.

## 1. Introduction

*Bovine alphaherpesvirus* 1 (BoHV-1) is a member of the *Herpesviridae* family, subfamily *Alphaherpesvirinae*, and belongs to the genus *Varicellovirus* [1]. BoHV-1 infection is associated with different clinical syndromes or symptoms such as infectious bovine rhinotracheitis (IBR), infectious pustular vulvovaginitis, infectious balanoposthitis, fever, dyspnoea, conjunctivitis, nasal discharge, abortions, enteritis, and encephalitis [2,3]. The virus is an important pathogen in cattle; its infection causes severe economic losses to the livestock industry worldwide, including international trade restrictions imposed by European IBR-free countries [4,5,6].

Recently, the European Union has issued new animal legislation, the so-called “Animal Health Law” [7], and several delegated regulations [8,9,10,11,12,13,14] that include IBR have been published. In particular, the Delegated Regulations (EU) 2020/688 [10] and 2020/689 [11] established diagnostic methods for granting and maintaining the IBR-free zone of IBR-free herds, using different tests on serum, milk, and meat juice samples. Enzyme-linked immunosorbent assay (ELISA) and virus neutralisation test (VNT) represent excellent diagnostic tools to detect IBR antibodies and identify seropositive animals [15,16]. VNT is considered the reference test for IBR and is a useful tool for comparing antibody titres in animals. However, ELISA tests are highly sensitive, easy to use, incur a low cost to obtain reliable results, do not require laboratories to handle live BoHV-1, and are suitable for screening large numbers of samples to estimate levels of IBR antibodies [17,18,19].

Commercial blocking gB-ELISAs or whole-virus-based indirect ELISAs with high diagnostic specificity (DSp > 95%) and diagnostic sensitivity (DSe > 99%) are commonly used to identify anti-BoHV-1 antibodies in serum and milk samples [15,20]. In particular, the commercial blocking ELISAs are used to detect specific antibodies against glycoprotein B (gB-ELISA) or gE (gE-ELISA) of BoHV-1, and they can be used in individual or pooled samples [21]. However, in IBR-free farms or marker-vaccinated herds, the blocking gE-ELISA only allows discrimination between infected and immunised animals with gE-deleted markers [21,22,23,24]. In the last 20 years, commercial blocking ELISAs, using both serum and milk samples, have been developed by different companies; however, the blocking ELISAs using milk samples are discouraged because of the chance of obtaining false positives [22]. Therefore, few diagnostic tests on milk samples are commercially available for the surveillance or eradication of IBR programs. Furthermore, the results obtained from gB-ELISA tests, in general, indicate the presence of specific antibodies against gB of BoHV-1 [25]; in some cases, this positivity could originate from other herpesviruses such as *Bovine alphaherpesvirus* 2 [26,27,28]. It has also been shown that positive results from gE-ELISAs in cattle or buffalo could be influenced by a previously circulating wild-type BoHV-1 in the herd [29,30]. In addition, to improve the sensitivity of blocking gE-ELISAs, some methods use milk-concentrating protocols [21,23,31,32].

Nevertheless, bulk milk (BM) testing could represent a useful tool for eradication and/or monitoring programmes because of the easier collection, lower sample collection costs, and reduced animal stress [22,24,33]. Recent studies have assessed the feasibility of using ELISA tests on BM samples to detect the antibodies for IBR control and estimate the BoHV-1 prevalence in a herd [21]. However, the testing protocols of maintaining the minimum number of samples in BM samples should be ensured because of the lower concentration of antibodies (0.6 mg/mL) compared to that in blood (10 mg/mL) [31,34,35].

Furthermore, the current protocol [36] is subject to many limitations, including the availability of international reference materials primarily for diagnostic tests on milk samples. In addition, the current economic insecurity is another limitation for developing new assays to control infectious diseases in farm animals [24]. Therefore, improvising and validating the available tools is important to identify the correct surveillance strategy for control of IBR.

This study aims to improve and validate a commercial indirect ELISA kit to detect BoHV-1-specific gE antibodies in BM samples of dairy cows.

## 2. Materials and Methods

### 2.1. gE-ELISA (ERADIKIT)

In this study, the gE indirect ELISA kit (ERADIKIT™ Bulk Milk Surveillance Kit PLUS, In3diagnostic, Torino, Italy) was used following manufacturer recommendations (Table 1). It was then compared to the protocols described in previous studies [22,33], as slight modifications were done. The net optical density (OD) of each sample was calculated by subtracting the OD value of the negative antigen (even columns) from that of the positive antigen (odd columns). The percentage of reactivity of each sample was calculated as the ratio between the net OD value of the sample and the net OD value of the positive control as follows:

$$Reactivity\;\left(\%\right)=\frac{\left(\mathrm{Net}\;\mathrm{OD}\;\mathrm{value}\;\mathrm{of}\;\mathrm{the}\;\mathrm{sample}\right)}{\left(\mathrm{Net}\;\mathrm{OD}\;\mathrm{value}\;\mathrm{of}\;\mathrm{the}\;\mathrm{positive}\;\mathrm{control}\right)}\times 100$$

According to the cut-off established by the assay manufacturers, samples with a reactivity greater than 40% were classified as positive, those with reactivity between 30% and 40% as doubtful, and those with a reactivity lower than 30% as negative.

### 2.2. Criteria for Assay Validation

In this study, the approach adopted to validate the indirect gE-ELISA test included the following criteria:

1. Repeatability
2. Analytical sensitivity (ASe)
3. Diagnostic sensitivity (DSe)
4. Diagnostic specificity (DSp)
5. Diagnostic accuracy

For these evaluations, we primarily referred to protocols [36,37] of the OIE Manual.

The outcomes of all tests were analysed quantitatively and then qualitatively, based on the cut-off values established by the assay manufacturers.

### 2.3. Reference Samples

In this study, all data analysed were collected as a part of the routine diagnosis and not for this experiment; therefore, according to the national legislation, ethics approval and written informed consent were not required.

A panel of negative and positive reference samples based on milk samples from farms or animals with a known history and IBR status was created to evaluate the performance of the gE indirect ELISA test.

The negative reference samples comprised 80 BM samples collected from 80 different herds in northern Italy (Piedmont Region) with official IBR-free status. The farms were certified by the regional public veterinary service in 2019 under the IBR European legislation 2004/558/CEE [38]. Each BM sample comprised a maximum of 50 milk samples.

The National Reference Centre for Infectious Bovine Rhinotracheitis Biobank (Perugia, Italy) provided a set of samples (serum and individual milk) collected during an IBR outbreak that occurred in central Italy in 2018. The outbreak involved a dairy herd of approximately 270 cows, for which no IBR vaccine had been used in the last seven years. Twelve 4-year-old lactating cows were selected from this herd and divided into three groups of four animals each. Serum and individual milk samples were collected from the three groups at 10, 21, and 63 days after the start of the outbreak (time 0, official confirmation of IBR outbreak). BoHV-1 antibodies in serum samples were evaluated by gB-ELISA (IDEXX IBR gB X3 Ab, Westbrook, ME, USA), gE-ELISA (IDEXX IBR gE Ab test), and VNT. In addition, whole-virus ELISA (IDEXX BHV1 Bulk Milk Ab test), gB-ELISA (IDEXX IBR gB X3 Ab), and gE-ELISA (IDEXX IBR gE Ab test) were used to evaluate the antibody concentration in the milk samples.

The protocols used were those described by the ELISA kit manufacturers, and VNT was performed according to the protocol reported in the OIE Manual of Diagnostic Tests and Vaccines for Terrestrial Animals, the “Infectious Bovine Rhinotracheitis/Infectious Pustular Vulvovaginitis (Chapter 3.4.11) [39]. The results are presented in Table 2.

Twelve positive individual milk samples were tested using the gE indirect ELISA kit (ERADIKIT). Based on the OD results, they were grouped into three categories: weak, medium, and high reactivity. Each positive individual milk sample was diluted at 1:10, 1:20, and 1:40 in certified IBR-free milk, thereby obtaining a total of 36 milk samples, which were used as the positive reference samples for this study (Table 3).

### 2.4. Repeatability

To evaluate within-laboratory repeatability (variation in results of replicates), each positive reference sample was tested in four replicates: two replicates within the same test run (same operator on the same day) and two replicates in different sessions (two operators on different days).

Within-run repeatability was assessed by calculating the coefficient of variability (CV) for each sample, obtained as the ratio between the standard deviation and the arithmetic mean of the two replicates within the same test run. Similarly, between-run repeatability was assessed by calculating the CV of four replicates for each sample.

Then, the average within-run CV and between-run CV were calculated for each reactivity group and dilution, respectively, and the corresponding 95% confidence intervals (CIs) were estimated.

Additionally, for reporting purposes, an agreement analysis of the qualitative results of the four replicates was conducted to quantify the repeatability in terms of the agreement of test results beyond chance. To achieve this, Gwet’s agreement coefficient (AC) was calculated with a 95% CI. Cohen’s kappa statistic (κ) is generally the first choice for estimating agreement. However, it is known to be influenced by the prevalence of the attribute under study and, in some cases, can yield unexpected results, known as the paradoxes of kappa [40]. Gwet’s AC, on the other hand, has greater stability as the prevalence of the attribute under study varies; thus, it is a more reliable tool in the cases of unbalanced distributions such as the one under examination [40,41].

### 2.5. Analytical Sensitivity (ASe)

To assess the assay ASe, the limit of detection (LOD) was conservatively defined as the maximum dilution at which 100% of replicates showed positive results.

### 2.6. Diagnostic Sensitivity (DSe) and Diagnostic Specificity (DSp)

Based on the cut-off established by the kit manufacturers, the qualitative results of the test, which were obtained from the reference sample panel, were dichotomised as negative and non-negative (doubtful or positive) and cross-tabulated in a 2 × 2 table according to the IBR status of the reference samples. Then, according to Chapter 2.2.5 of the OIE Manual of Diagnostic Tests and Vaccines for Terrestrial Animals [37], estimates of the assay DSe (proportion of positive reference samples that tested positive in the assay) and of the assay DSp (proportion of negative reference samples that tested negative in the assay) were obtained, calculating the corresponding exact binomial 95% CIs.

### 2.7. Diagnostic Accuracy

To evaluate the diagnostic accuracy of the assay, agreement analysis was conducted to quantify the concordance between the qualitative results of the indirect ELISA and the true IBR status of the reference samples. Weighted Cohen’s kappa was computed, providing the respective 95% CI.

### 2.8. Statistical Analysis: Tools and Settings

Statistical analyses were conducted using Microsoft Excel 2013 (Microsoft Corporation, Redmond, WA, USA) and Stata^®^ 16.1 statistical software (Special Edition; StataCorp LP, College Station, TX, USA). The significance level was set at α = 0.05.

## 3. Results

### 3.1. Repeatability and ASe

The quantitative results of the four replicates are shown in Table 4. Replicates I and II denote the two replicates performed in the same test run, and replicates III and IV denote the replicates performed in two different sessions. All samples were confirmed to be positive in all replicates except for the 1:40 diluted sample of ID-37, which was classified as doubtful (S/P%: 33.9).

**Figure 1:**
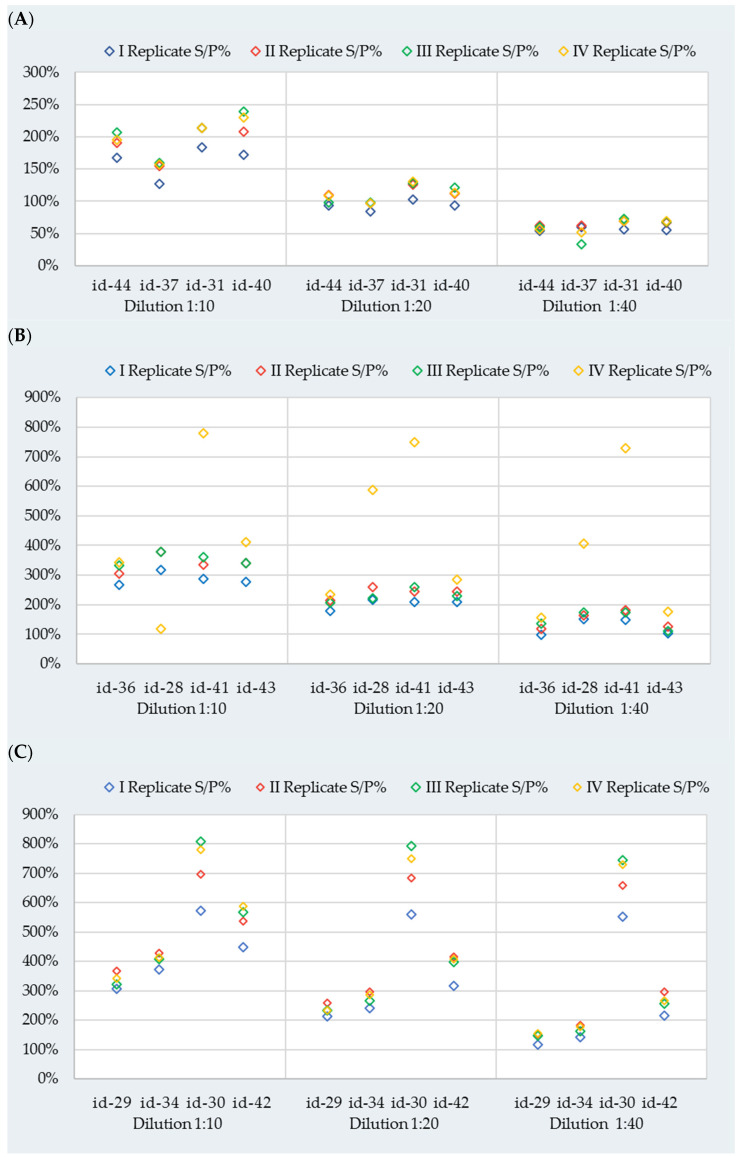
Results obtained from gE indirect ELISA expressed as S/P (positive-to-control ratio); (**A**) Weak reactivity positive samples, diluted at 1:10, 1:20, and 1:40, assayed in four replicates; (**B**) Medium reactivity positive samples, diluted at 1:10, 1:20, and 1:40, assayed in four replicates; (**C**) Strong reactivity positive samples, diluted at 1:10, 1:20, and 1:40, assayed in four replicates.

The assay showed a LOD of 1:20 for the weakly positive samples. In contrast, the assay LOD for the medium–highly positive samples was estimated to be at least 1:40 (Table 4; Figure 1).

**Figure 2:**
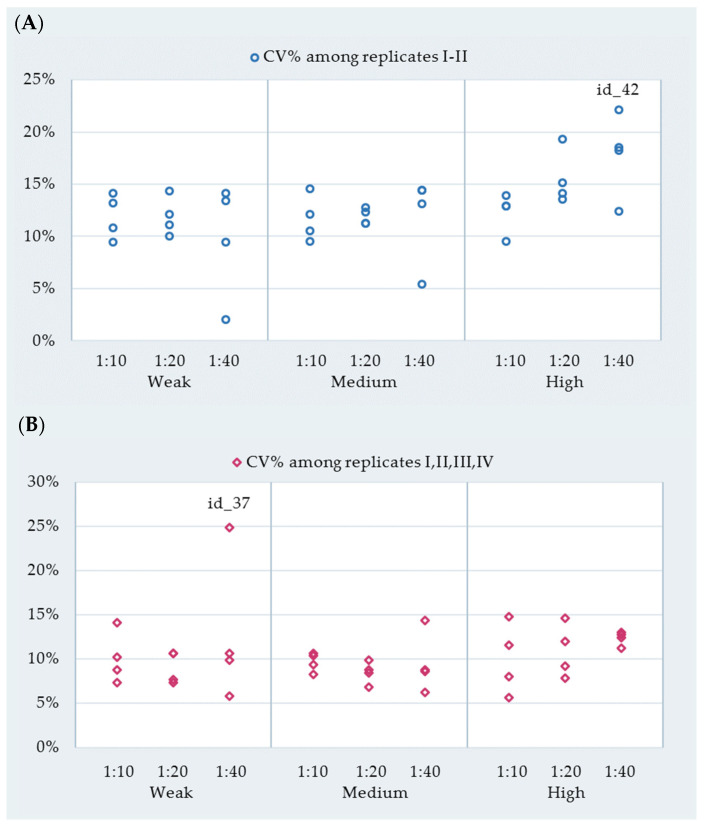
(**A**) Within-run coefficient of variability (CV%) among replicates I–II performed on each positive reference sample per reactivity level and dilution ratio. (**B**) Between-run coefficient of variability (CV%) among replicates I–IV performed on each positive reference sample per reactivity level and dilution ratio. For each reactivity group and dilution, there are four dots. Each dot represents the CV% among the replicates, performed on a single positive reference sample belonging to that reactivity level and originating from that dilution. The highest values of CV% were labelled as follows to aid communication: the CV% of a 1:40 diluted id-42 individual milk and the CV% of a 1:40 diluted id-37 individual milk.

The within-run and between-run variabilities expressed as the CV% of replicates I and II (I–II) and the CV% of replicates I, II, III, and IV (I–IV), respectively, are shown at the sample level in Table 5 and Figure 2.

The within-run CV% ranged between 2.0% and 22.1%. The CV% of one sample (ID-42; 1:40 diluted) exceeded 20% (Figure 2A). The between-run CV% ranged between 5.6% and 24.9%; nevertheless, only one sample (ID-37; 1:40 diluted) exceeded 15% (Figure 2B).

**Figure 3:**
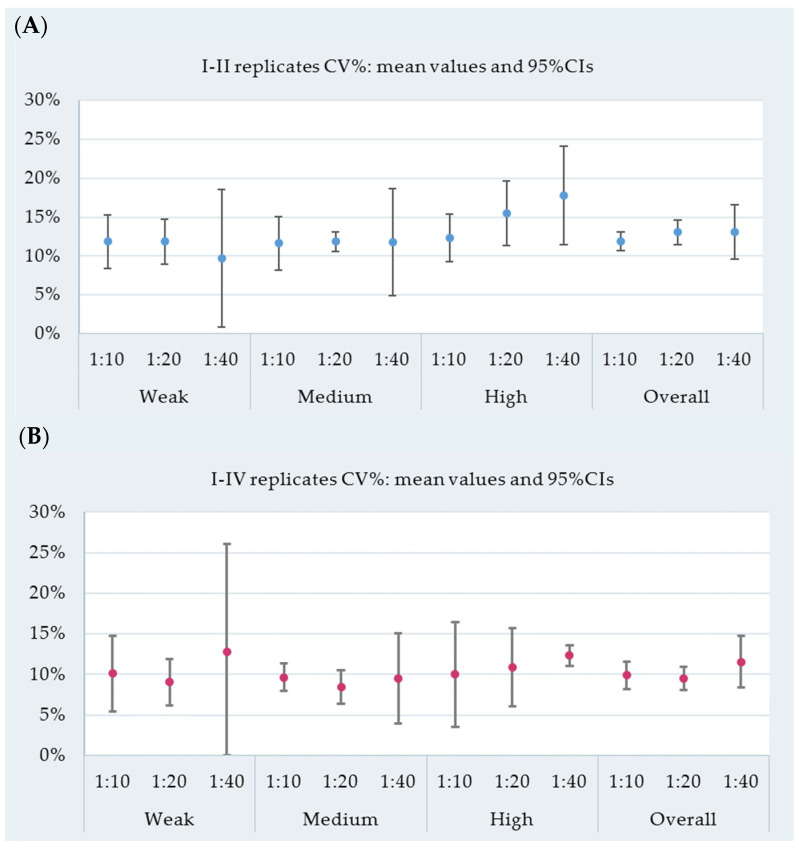
(**A**) Within-run coefficient of variability (CV%): mean value and 95% CI for each reactivity level and dilution. (**B**) Between-run coefficient of variability (CV%): mean value and 95% CI for each reactivity level and dilution ratio.

For each reactivity group and dilution ratio, the mean value of within-run CV% and the mean value of between-run CV% are shown in Figure 3, respectively, with corresponding 95% CIs. Additionally, overall averages of CV% were computed for each dilution, regardless of reactivity.

On average, the within-run variability for each reactivity level and dilution did not exceed 20%, except for highly reactive samples at a 1:40 dilution (CV%: 17.8, 95% CI: 11.4–24.2; Figure 3A). Overall, at 1:10 and 1:20 dilutions, the assay within-run CV% ranged from 10% to 15% on average (1:10 dilution CV%: 11.9, 95% CI: 10.7–13.2; 1:20 dilution CV%: 13.1, 95% CI: 11.5–14.7); among 1:40 diluted samples, the average CV% was 13.1% (95% CI: 9.6–16.6; Figure 3A).

The between-run repeatability for each reactivity level and dilution did not exceed 16.4%, except for weakly reactive samples at a 1:40 dilution (CV%: 12.8, 95% CI: 0.0–26.1; Figure 3B). Overall, the between-run repeatability CV% ranged from 8% to 15% on average (1:10 dilution CV%: 9.9, 95% CI: 8.2–11.6; 1:20 dilution CV%: 9.5, 95% CI: 8.1–10.9; 1:40 dilution CV%: 11.6, 95% CI: 8.4–14.7; Figure 3B).

The agreement analysis on the qualitative results of the four replicates led to a Gwet’s AC of 0.99 (95% CI: 0.96–1, *p* < 0.001), which, according to Altman’s scale, corresponds to a ‘very good’ agreement [42].

### 3.2. Diagnostic Performances: DSe, DSp, and Diagnostic Accuracy

**Figure 4:**
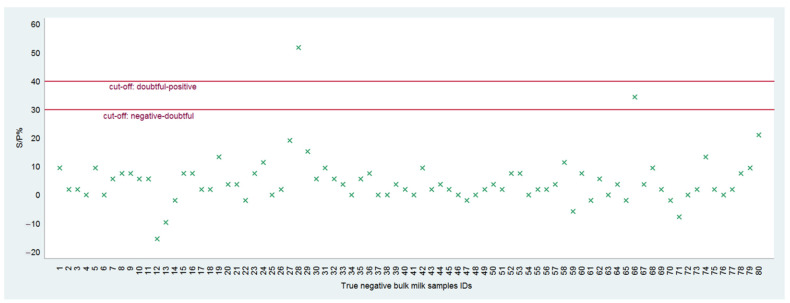
Results obtained from the negative reference bulk milk samples tested with the gE indirect ELISA.

Overall, 78 out of 80 negative reference samples were confirmed as negative by gE indirect ELISA (Figure 4). Only 2 samples out of 80 generated false-positive reactions (one positive: S/P, 52.8%; one doubtful: S/P, 34.6%), although they were classified as negative when tested a second time.

The qualitative results of the gE indirect ELISA performed on the 116 reference samples were dichotomised as negative and non-negative and cross-tabulated according to the IBR status of the samples (Table 6). The assay showed a DSe of 100% (95% CI: 90.3–100%) and a DSp of 97.5% (95% CI: 91.3–99.7%).

Additionally, to quantify the overall ability of the assay to correctly categorise samples, the agreement analysis of the 116 reference samples led to a weighted Cohen’s κ of 0.96 (95% CI: 0.78–1; Table 7). According to Altman’s scale, a value of κ ranking between 0.61 and 0.80 corresponds to a ‘good’ agreement, while a value of κ higher than or equal to 0.81 corresponds to a ‘very good’ agreement [42].

## 4. Discussion

The improvisation and validation of the available assays is a prerequisite for dealing with current economic insecurities. Furthermore, all assays should be validated to monitor their routine performance for an intended purpose. Validation is a process that includes estimates and optimisation of all analytical and diagnostic performance characteristics of a test [36]. Several commercial gE indirect ELISA kits intended to detect BoHV-1 in BM samples have been developed [20,22,23,33]. For their application in a screening test in a surveillance programme, they should be capable of detecting all levels of true positivity, especially those borderline cases where animals have low antibody levels.

In this study, we made concerted efforts to improve and validate the performance of a commercial gE indirect ELISA kit: the ERADIKIT™ Bulk Milk surveillance Kit PLUS. The protocol for the gE indirect ELISA test was applied to skimmed milk and was modified by modifying the steps for the purification and concentration of IgG to enhance the kit’s performance compared to the original protocol described in Muratore et al. [33].

The protocol described earlier [33] only evaluated DSe, DSp, and ASe, and was successful.

Here, we modified the DSe (100%; 95% CI: 90.3–100%), DSp (97.5%; 95% CI: 91.3–99.7%), incubation times, room temperatures, washing step, sample number and volumes, dilution factors, and wash buffer formulation of the original protocol to ensure the fitness of the kit for the intended purpose. The protocol developed herein validated the results of the original protocol [33]. It showed similar results compared with those obtained using the original protocol (DSe = 100%; 95% CI: 89.4–100% and DSp = 98.85%; 95 CI: 95.90–99.86%). However, CIs, which indicate the precision of the DSe and DSp estimates, were wide. This could be attributed to the limited number of samples used to estimate the sensitivity and specificity, as it is difficult to find field samples with well-defined IBR status. Furthermore, the optimisation of the reagents and protocol by including samples belonging to animals with known infection status that had a definite level of activity (titre or concentration) for the pathogen could also have affected the results.

In this study, we demonstrated good agreement between the test results and those of the IBR status of samples, repeatedly achieving the same results for the milk samples examined. The evidence of adequate within-laboratory repeatability was highlighted by raw absorbance values of less than 20%, meeting the requirements of the OIE Manual of Diagnostic Test and Vaccines for Terrestrial Animals (CV < 30%) [43,44]. Only two samples generated false-positive reactions, with values of CV% approaching 20–30%, but they were restored when tested a second time. It could be due to an unavoidable minimum bias and random error [44].

Collectively, in this study, calculating beforehand the prevalence within each BM sample [45] using the preliminary knowledge of reactivity of each positive serum individual sample demonstrated more precision of the DSe and DSp estimates and adequate repeatability of the assay compared to those obtained by Muratore et al. [33]. Moreover, as described in previous studies [23], the antibody titre and dilution factor also partially influenced the analytical sensitivity in the present study. The assay ASe showed a LOD of 1:20 for the weakly positive samples compared to at least 1:40 for the medium–highly positive samples. Therefore, even if some steps for IgG purification and concentration processes were modified, the indirect gE-ELISA partially detected weakly positive samples in concentrated pooled milk samples [19,23]. Several studies have demonstrated that weakly positive samples are frequently missed before dilution [15,23]. Therefore, it has been suggested to check the performance of the competitive ELISA tests using various dilutions to identify the maximum dilution at which 100% of replicates can improve the positive results. Moreover, as suggested by Schroeder et al. [23], reducing the pool size from 50 to 20 milk samples or establishing regular testing (e.g., monthly) might improve the precision of the results. In such a case, all 1:20 dilutions of weakly positive samples would have been detected by the assay validated in this study.

In addition, several other points must be taken into account while using the indirect ELISA to detect BoHV-1–specific gE antibodies in BM samples.

Increasing the IgG concentration in milk samples to increase the sensitivity of the test is a major drawback [23]. This procedure requires a longer period for serological analysis and the use of more laboratory materials.

Moreover, under the Delegated Regulation (EU) 2020/689, some prerequisites of BoHV-1 BM testing must be satisfied. In particular, a pool of no more than 50 milk samples (individual or BM) may be used in tests for granting IBR-free status, and a pool containing no more than 100 milk samples (individual or bulk) may be used for the maintenance of IBR-free status [11]. Additionally, serological tests against gE of BoHV-1 must be performed on BM samples from farms with at least 30% of the lactating bovine. In this case, milk samples must be taken on at least three occasions at intervals of not less than three months from lactating bovine representing all epidemiological units of the herd. Moreover, blood samples must be collected from all non-lactating bovines and male bovines over 12 months of age. In a herd comprising less than 5% male bovines wherein at least 95% of females used for milk production are over 24 months of age, BM samples must be taken on at least six occasions at intervals of not less than 2 months from lactating animals representing all epidemiological units of the establishment [11].

However, milk sampling represents a fast and non-invasive procedure for animals because it avoids negative effects, such as stress, and reduces the total cost of surveillance [46]. In addition, such samples can be easily collected, pooled, and tested. This can increase the number of serological investigations per year, allowing earlier detection of new BoHV-1 outbreaks [22,33]. Therefore, further studies are required to improve the validation pathway of this innovative serological kit, such as the use of European reference materials collected from cattle and buffalo.

## 5. Conclusions

In conclusion, the results of this study indicate that the new protocol gE indirect ELISA test “ERADIKIT™ Bulk Milk Surveillance Kit PLUS” did not improve the kit’s performance significantly compared to the original protocol described by Muratore et al. [33]. However, the new procedure could be considered a useful tool, provided that the applicability of indirect gE-ELISA testing of IgG concentration in milk samples could be employed in a pool of individual samples from no more than 40 lactating cows, where individual samples are available for routine analysis (i.e., somatic cell count, protein, and fat content), as suggested by Colitti et al. [22]. Furthermore, the applicability of gE-ELISA for BM testing, without the IgG concentration step, proved to be suitable to detect BoHV-1 in vaccinated herds, provided that gE prevalence is higher than 10%. Nevertheless, in this case, more frequent testing is necessary, so that most cows present in a herd will contribute to a BM sample at least once per year [20]. Further studies are required to evaluate the gE indirect ELISA test “ERADIKIT™ Bulk Milk Surveillance Kit PLUS” using European reference materials collected from cattle and buffalo.

## 6. Patents

The patent application was registered using the In3diagnostic (EP 2,942,628 A1 Kit and in vitro method for identifying BoHV-1 infection).

## Figures and Tables

**Table 1:** Main differences between the protocols used in the gE indirect ELISA described by Muratore et al. (2017) (gE I) [33], reported by Colitti et al. (2018) (gE II) [22], and used in this study (gE III).

Process Steps		Protocols		
		gE I	gE II	gE III
	**Diagnostic Matrix**	**Whole Milk**	**Whole Milk**	**Skimmed Milk**
**Purification and concentration of IgG**	**Incubation**	5 min on ice with rennet-based casein precipitation	20 min at 37 °C with precipitating reagent A; curd broken by manual agitation and incubated on ice for 10 min	10 min at 37 °C followed by 50 min with precipitating reagent A, always in a water bath
	**Centrifugation**	3600× *g* for 10 min at 4 °C	3600× *g* for 10 min at 4 °C	2500× *g* for 15 min at 23 ± 3 °C
	**Separation from lipids and curd**	6 mL milk with affinity matrix for 10 min	7 mL whey added with equal volume of precipitating reagent B; 60 min incubation on a platform shaker at 23 ± 3 °C	7 mL whey milk added with equal volume of precipitating reagent B; 30 min incubation on a platform shaker at 23 ± 3 °C
	**Centrifugation and wash**	Centrifuge and wash twice	3600× *g* for 10 min at 18 ± 3 °C and tube upside down (1–2 min)	2500× *g* for 20 min at 23 ± 3 °C and tube upside down (1–2 min)
	**Elution**	In 200 μL glycine buffer	In 400 μL serum dilution buffer	In 800 μL serum dilution buffer
	**Confirm the presence of IgG**	Bradford Quantification assay Bovine Gamma Globulin (BIORAD Quick Start) standard curve	Once a compact pellet becomes visible, detach it and vortex to obtain a consistent solution	Once a compact pellet becomes visible, detach it and vortex to obtain a consistent solution
**ELISA**	**Volume sample (μL)**	Dilution 1:2 in phosphate-buffered saline (PBS) containing 1.25% casein	200 μL IgG concentrated sample	200 μL IgG concentrated sample
	**Incubation**	60 min at 23 ± 3 °C	2 h at 23 ± 3 °C	16 ± 2 h at 2–8 °C
	**Addition of peroxidase-labelled secondary antibody**	Diluted at 10 ng/mL in PBS containing 1.25% casein	Diluted at 10 ng/mL in PBS containing 1.25% casein	Before use, dilute the 100 X conjugate in conjugate dilution buffer

**Table 2:** Results obtained from serum and individual milk samples collected from twelve 4-year-old lactating cows during an IBR outbreak that occurred in 2018 in central Italy.

Group	Cow ID	Samples					
		Serum			Individual Milk		
		gE-ELISA ^d^	gB-ELISA ^e^	VNT ^f^	Whole-Virus ELISA ^g^	gE-ELISA ^d^	gB-ELISA ^e^
1 ^a^	31	+	+	1:512	+	+	+
	37	+	+	1:512	+	+	+
	40	+	+	1:256	+	+	+
	44	+	+	1:256	+	+	+
2 ^b^	28	+	+	1:1024	+	+	+
	36	+	+	1:2048	+	+	+
	41	+	+	1:1024	+	+	+
	43	+	+	1:1024	+	+	+
3 ^c^	29	+	+	1:2048	+	+	+
	30	+	+	1:4096	+	+	+
	34	+	+	1:4096	+	+	+
	42	+	+	1:4096	+	+	+

^a^ Collected 10 days after the start of the outbreak; ^b^ Collected 21 days after the start of the outbreak; ^c^ Collected 63 days after the start of the outbreak; ^d^ IDEXX IBR gE Ab test, Maine, USA; ^e^ IDEXX IBR gB X3 Ab, Maine, USA; ^f^ VNT, virus neutralisation test; ^g^ IDEXX BHV1 Bulk Milk Ab test.

**Table 3:** Positive individual milk samples tested with ERADIKIT™ BMSK PLUS and diluted respectively at 1:10, 1:20, and 1:40 to obtain the set of positive reference samples.

Cow ID	OD Values ^a^			S/P% ^e^	Reactivity	Dilutions Performed	No. of Positive Reference Samples
	Ag+ ^b^	Ag– ^c^	Net ^d^				
44	1.314	0.155	1.159	264.6	Weak	1:10 1:20 1:40	4 4 4
37	1.491	0.262	1.229	280.6			
31	1.581	0.213	1.368	312.3			
40	1.616	0.158	1.458	332.9			
36	2.322	0.2	2.122	484.5	Medium	1:10 1:20 1:40	4 4 4
28	2.492	0.279	2.213	505.3			
41	2.703	0.46	2.243	512.1			
43	2.545	0.166	2.379	543.2			
29	2.985	0.272	2.713	619.4	High	1:10 1:20 1:40	4 4 4
34	3.002	0.25	2.752	628.3			
30	4.029	0.958	3.071	701.1			
42	3.467	0.206	3.261	744.5			
Total of positive reference samples							36

^a^ OD—optical density; ^b^ Ag+—well of the plate with antigen; ^c^ Ag–—well of the plate without antigen; ^d^ Net—OD_Ag+_ minus OD_Ag-_; ^e^ S/P%—sample-to-positive control ratio.

**Table 4:** Results obtained from gE indirect ELISA using positive reference samples assayed in four replicates.

Reactivity	Cow ID	Dilution	Replicates (S/P%)			
			I	II	III	IV
Weak	id-44	1:10	167.3	191.1	207.1	195.0
	id-37		126.9	154.9	159.2	157.1
	id-31		183.6	214.0	213.3	213.9
	id-40		172.1	207.7	239.8	229.5
	id-44	1:20	93.8	109.8	98.0	108.7
	id-37		83.8	96.6	98.7	97.2
	id-31		102.9	126.2	128.8	130.4
	id-40		94.0	111.5	121.7	113.4
	id-44	1:40	54.7	62.6	59.7	56.8
	id-37		60.6	62.3	33.9	52.4
	id-31		56.5	68.9	72.4	69.3
	id-40		55.6	67.2	68.4	69.6
Medium	id-36	1:10	268.0	306.4	332.1	341.7
	id-28		318.8	378.3	379.3	380.0
	id-41		288.8	335.3	359.9	365.6
	id-43		277.5	340.6	340.1	330.4
	id-36	1:20	179.3	213.4	207.7	220.5
	id-28		217.7	260.6	223.2	245.5
	id-41		208.4	244.3	258.7	260.1
	id-43		208.4	244.3	229.6	238.7
	id-36	1:40	98.6	118.7	136.2	135.1
	id-28		151.1	163.2	174.2	170.3
	id-41		148.0	181.7	174.5	170.3
	id-43		103.4	126.8	110.2	117.7
High	id-29	1:10	306.2	367.7	321.7	342.2
	id-34		373.7	427.7	407.7	412.3
	id-30		572.3	696.8	808.2	779.2
	id-42		447.5	537.0	568.6	586.8
	id-29	1:20	213.1	257.9	233.9	233.5
	id-34		239.9	297.2	266.8	286.1
	id-30		559.7	683.4	793.4	749.1
	id-42		316.4	416.6	397.4	407.3
	id-29	1:40	116.9	152.1	148.0	155.7
	id-34		141.5	183.2	163.3	177.4
	id-30		552.8	659.1	744.6	728.5
	id-42		216.7	297.0	257.1	266.7

S/P: sample-to-positive control ratio.

**Table 5:** Evaluation of within-run repeatability and between-run repeatability on the positive reference samples, showing mean value, standard deviation, and coefficient of variation of S/P (sample-to-positive control ratio) among replicates (I–II and I–IV respectively) for each positive reference sample.

Reactivity	Cow ID	Dilution	Replicates I–II			Replicates I–IV		
			Mean *	SD ^‡^	CV%	Mean *	SD ^‡^	CV%
Weak	id-44	1:10	179.2	16.8	9.4	190.1	16.7	8.8
	id-37		140.9	19.8	14.1	149.5	15.2	10.2
	id-31		198.8	21.5	10.8	206.2	15	7.3
	id-40		189.9	25.1	13.2	212.3	29.9	14.1
	id-44	1:20	101.8	11.3	11.1	102.6	7.9	7.7
	id-37		90.2	9	10	94.1	6.9	7.3
	id-31		114.5	16.4	14.3	122.1	12.9	10.6
	id-40		102.7	12.4	12.1	110.1	11.7	10.6
	id-44	1:40	58.6	5.5	9.4	58.5	3.4	5.8
	id-37		61.5	1.2	2	52.3	13	24.9
	id-31		62.7	8.8	14.1	66.8	7.1	10.6
	id-40		61.4	8.2	13.4	65.2	6.5	9.9
Medium	id-36	1:10	287.2	27.2	9.5	312.1	33	10.6
	id-28		348.5	42.1	12.1	364.1	30.2	8.3
	id-41		312.1	32.9	10.5	337.4	35	10.4
	id-43		309	44.7	14.5	322.1	30.2	9.4
	id-36	1:20	196.4	24.1	12.3	205.2	18	8.8
	id-28		239.2	30.3	12.7	236.8	19.9	8.4
	id-41		226.3	25.3	11.2	242.9	24.1	9.9
	id-43		226.3	25.3	11.2	230.2	15.7	6.8
	id-36	1:40	108.7	14.2	13.1	122.2	17.6	14.4
	id-28		157.2	8.5	5.4	164.7	10.1	6.2
	id-41		164.9	23.8	14.4	168.6	14.5	8.6
	id-43		115.1	16.5	14.4	114.5	10	8.8
High	id-29	1:10	336.9	43.5	12.9	334.4	26.6	8
	id-34		400.7	38.2	9.5	405.3	22.8	5.6
	id-30		634.5	88	13.9	714.1	105.7	14.8
	id-42		492.3	63.3	12.9	535	61.8	11.6
	id-29	1:20	235.5	31.7	13.5	234.6	18.3	7.8
	id-34		268.6	40.5	15.1	272.5	25.1	9.2
	id-30		621.6	87.5	14.1	696.4	101.7	14.6
	id-42		366.5	70.9	19.3	384.4	46.1	12
	id-29	1:40	134.5	24.9	18.5	143.2	17.8	12.4
	id-34		162.3	29.5	18.2	166.3	18.6	11.2
	id-30		606	75.2	12.4	671.3	87.2	13
	id-42		256.9	56.8	22.1	259.4	33.2	12.8

* mean value of S/P% among replicates I–II and among replicates I–IV;. ^‡^ SD: standard deviation of S/P% among replicates I–II and replicates I–IV, respectively; CV: coefficient of variation of S/P% among replicates I–II and replicates I–IV, respectively.

**Table 6:** Dichotomised results of gE indirect ELISA performed on the reference samples panel: cross-tabulation according to the IBR status of the reference samples.

Test Results	Reference Samples		Total
	True Positive	True Negative	
Non-negative *	36	2	38
Negative	0	78	78
Total	36	80	116

* Non-negative: positive or doubtful result.

**Table 7:** Qualitative results of ERADIKIT™ BMSK PLUS obtained using the reference samples panel: cross-tabulation according to the true IBR status of the reference samples.

Test Results	Reference Sample Panel			Total
	True Positive	Doubtful	True Negative	
Positive	35	0	1	36
Doubtful	1	0	1	2
Negative	0	0	78	78
Total	36	0	80	116

## Data Availability

All datasets are included in the article.
